# In Vitro Evaluation of Bioavailability of Se from Daily Food Rations and Dietary Supplements

**DOI:** 10.3390/nu15061511

**Published:** 2023-03-21

**Authors:** Piotr Bawiec, Jan Sawicki, Paulina Łasińska-Pracuta, Marcin Czop, Ireneusz Sowa, Katarzyna Iłowiecka, Wojciech Koch

**Affiliations:** 1 Department of Food and Nutrition, Medical University of Lublin, 4a Chodźki Str., 20-093 Lublin, Poland; 2 Department of Analytical Chemistry, Medical University of Lublin, 4a Chodźki Str., 20-093 Lublin, Poland; 3 Department of Clinical Genetics, Medical University of Lublin, Radziwiłłowska 11 Str., 20-080 Lublin, Poland

**Keywords:** trace elements, selenium, diets, bioavailability, ICP-OES

## Abstract

Bioavailability refers to a fraction of a substance that is potentially absorbed from the gastrointestinal tract and enters the systemic circulation (blood). This term is related to various substances, including minerals, that are present in a complex matrix of food which is consumed every day as natural products and pharmaceutical preparations, e.g., dietary supplements. The purpose of the study was to assess the bioavailability of Se from selected dietary supplements, with the simultaneous assessment of the effect the diet type (standard, basic and high-residue diets) has on relative bioavailability. The research included a two-stage in vitro model of digestion using cellulose dialysis tubes of the food rations with the addition of dietary supplements. Se was determined using the ICP-OES method. The bioavailability of Se from dietary supplements, in the presence of food matrix, was determined to be within the range of 19.31–66.10%. Sodium selenate was characterized by the highest value of this parameter, followed by organic forms and sodium selenite. The basic diet, characterized by moderate protein and high carbohydrate and fiber contents, positively influenced the bioavailability of Se. The bioavailability of Se was also influenced by the pharmaceutical form of the product—the highest was for tablets, followed by capsules and coated tablets.

## 1. Introduction

Trace elements are important parts of nutrients and, therefore, are essential to humans. Their major source is food, including animal- and plant-derived foods of natural origin [1,2]. However, in recent years, the role of drugs, but above all dietary supplements, in the total intake of trace elements began to increase very quickly, as a result of which such products should be considered an important complement to the daily natural diet, which has not only nutritional but also significant socio-economic importance [3,4]. Studies on the concentration of various elements in food products and estimation of their dietary intake using direct chemical analysis have been performed for many years. They are important when it comes to determining element concentrations in various foods and reliable levels of intake [5,6,7,8,9]. However, considering the significance of trace elements for human health, not only the amount consumed but also the bioavailability—the actual amount of a substance that reaches the systemic circulation (blood) and can cause an effect in the body—is of utmost importance [10,11]. In the case of trace elements in food and soil samples, oral bioaccessibility is very often estimated; oral bioaccessibility is a maximum fraction of a substance theoretically released in the gastrointestinal (GI) tract from the food matrix (bioaccessible fraction) that is available for absorption [12].

An estimation of oral bioavailability and bioaccessibility of particular elements can be performed using in vivo and in vitro models. Both these parameters are affected by the type of food matrix and the chemical formula of particular compounds. However, the physiological condition of the GI tract and many other parameters, such as age, gender and state of health, are also very important parameters affecting the level of bioavailability and can be evaluated only by using a specific human model. Because of ethical considerations, especially when bioavailability is assessed for toxic elements (Hg, Pb or Cd), such practices are considered unethical [10,13]. In vitro methods using simulated GI digestion can imitate various parameters of the digestive tract (temperature, agitation, pH, enzyme composition). They are very easy and cheap to perform and are free of ethical concerns. Therefore, these methods are very often used for preliminary estimations of the level of bioavailability of elements from food products, including dietary supplements [10]. Such methods mimic the GI tract parameters and take into account complex interactions between nutrients, food matrix, pH, methods of food processing and preservation, etc. There are many various modifications of these methods, including the evaluation of a bioaccessible fraction [10], the estimation of a bioaccessible fraction using human GI microbiota cells [14,15], the estimation of a dialyzable fraction of the compound that can dialyze through semi-permeable cellulose membranes with specific pore sizes (bioavailable fraction), imitating the human intestine [16,17,18], and studies using human Caco-2 cells [19]. Although each of these methods has some specific limitations, their lack of ethical concerns, coupled with relative simplicity and the possibility of introducing various modifications, makes them widely used in the assessment of trace element bioavailability from foods, dietary supplements and different pharmaceutical products [17,18,20]. Although all in vitro methods based on chemical experiments do not reflect the typical rate of entry into the circulation, in our opinion, it seems that methods using cellulose membranes reflect the natural conditions in the GI tract to a greater extent, which is why we decided to use such a methodology, and not only the assessment of the bioaccessible fraction.

Selenium (Se) essentiality to humans has been widely known for over 60 years [21], and its role is mainly associated with its presence in the active center of various antioxidant enzymes, making Se a crucial element in maintaining proper redox balance in the body [22,23,24]. Se is also involved in the regulation of thyroid function, skin development and heavy metal detoxification [22,25,26]. It was also suggested that higher Se exposure through diet or supplementation protects humans against several types of cancers. However, the results of different clinical trials are inconclusive, and there is no evidence that Se may be considered as an anticancer agent [27]. An optimal range of Se intake is very narrow, from 60 to 70 µg/day in various countries [22], and the latest recommended dietary allowance (RDA) for this element in Poland for people over the age of 13 has been established at 55 µg/day, for both women and men [28]. Se content in food products is strictly correlated with its concentration in the soil, and its deficiency in humans causes brittle hair and nails and serious cardiac, thyroid and osteoarthritic conditions [22,25,26,29,30]. In contrast, high Se intake (mostly through excessive supplementation) may cause severe intoxication; thus, the amount which is consumed with food and dietary supplements should be strictly controlled [22]. Se enters the body only through the diet, and the amount which is available to the organism is correlated to the dose and chemical form in which the element is consumed [22,31,32]. Recent studies revealed that Se intake in Poland varies within a wide range from 36.9 to 106 and from 64.7 to 119 µg/day among women and men, respectively [5,6]. For the majority of the population, it is higher than the recommended RDA standard; however, there are groups of people characterized by low dietary intake and low Se levels in blood and urine [33]. For such people, Se supplementation seems to be justified. Despite the lack of official recommendations, Se supplementation in a preferable dose of 100–200 µg/day is recommended by the majority of European endocrinologists for autoimmune thyroiditis (AIT) patients. However, there are no official guidelines for oral Se supplementation, and U.S. Food and Drug Administration (FDA) has not approved any oral preparation containing Se [34].

Se in food products is present in a variety of chemical forms, which in general are divided into organic and inorganic [32]. There are data indicating that, depending on the type of a food product, there is a predominance of a specific form of this element. Organic forms, predominantly selenomethionine and selenocysteine, are dominant forms in fish and animal products [35,36]. Inorganic forms of Se (selenite and selenate) are important in the biological and biochemical cycles of Se and are well absorbed by plants; thus, they can be found in food of plant origin [37]. The biochemical properties of selenite and selenate are different, including their toxicity and energy consumption during uptake and metabolism [37,38]. In general, organic forms of Se are considered more effective for increasing endogenous human and animal levels and less toxic than inorganic forms of Se [32,37,39]. Both the organic and inorganic forms of the element are widely used in the form of dietary supplements and other nutritional sources of Se, such as functional foods, but they are all toxic in higher doses; thus, the intake of Se should be strictly monitored [37]. Inorganic forms of Se (sodium selenite and selenate) are mostly used in the production of multi-vitamin/mineral dietary supplements, and organic forms (L-selenomethionine or selenium-enriched yeast) are preferably used for the production of supplements that provide only Se. However, it should be remembered that the market of supplements is not regulated and producers are free to choose and adjust the composition of their production, of course in compliance with current law [40].

The bioavailability of Se depends on the type of food product and the method of thermal processing of food. Fish are considered a very good dietary source of Se because the content of the element and its bioavailability from these products are very high [32]. Cabanero et al. [41] investigated the bioavailability of Se from commonly consumed fish in Europe using an in vitro method. This study revealed that fish are a very good dietary source of Se in a daily human diet. The determined bioavailability of Se was 80% from sardine, 76% from swordfish and 50% from tuna. Similar results were obtained by Singhato et al. [32] who determined the bioavailability of Se from various fish commonly consumed in Thailand, which was 58.8% for boiled and 51.1% for fried fish. No influence of the cooking practice on the bioavailability of Se was revealed. Moreover, fish were characterized as a very rich dietary source of Se containing up to 198.5 µg of Se per 100 g of a fresh, edible portion. Such data confirm that natural diets, rich in high-Se-content foods, should be the most important sources of Se for the human body.

The majority of studies on the issue of the bioavailability of Se focus on the influence of its chemical form, dose and differences between various food products. However, in the scientific literature, there is a lack of data on the influence of whole, complex diets on the absorption of Se from dietary supplements. The type of pharmaceutical preparations (tablets, capsules) and their influence on bioavailability were not evaluated either. Therefore, the main purpose of the research was to assess the relative bioavailability of Se from dietary supplements and medicinal products, with the simultaneous assessment of the influence of the diet type on relative bioavailability. In addition, the influence of the chemical forms of given elements and the pharmaceutical forms of tested preparations on the effectiveness of their bioavailability was assessed. Taking into account the fact that a balanced diet should include products from all food groups, reconstructed daily food rations (DFRs) in the form of homogenates consisting of four meals were used in the study. As it was mentioned above, in vitro methods of bioavailability evaluation of various minerals, using a simulated digestion process and cellulose dialysis tubes, are widely employed [18,32,42,43].

## 2. Materials and Methods

The results of the present research are based on chemical determinations of bioavailable fractions of Se from dietary supplements in the presence of different types of diets after simulated digestion using the equilibrium dialysis technique and the ICP-OES method. The latter analytical technique was used as the most preferable due to its low detection limits and minimization of interferences. To the best of our knowledge, this is the first study to evaluate the effect of the type of diet and the pharmaceutical form of the product on the bioavailability of Se.

Although the term bioaccessibility refers to a fraction of a compound that is ready to be absorbed and the term bioavailability refers to a portion that is absorbed into the circulation [44], both these terms are used interchangeably in the scientific literature. Because the term bioavailability is the most frequently used in scientific papers for in vitro studies in which cellulose dialysis tubes are used to filtrate a digested fraction [17,18,45,46], we also used this term in the present study.

### 2.1. Chemicals and Reagents

Nitric acid, hydrochloric acid and hydrogen peroxide were of Suprapur grade and were bought from Merck (Darmstadt, Germany). A standard solution of Se (1 mg/L) was bought from PlasmaCAL (SCP SCIENCE, Baie-D’Urfé, QC, Canada). The enzymes used during simulated digestion (pepsin and pancreatin) and the dialysis tubes were purchased from Sigma-Aldrich (St. Louis, MO, USA). Sodium bicarbonate was bought from Avantor Performance Materials (POCH, Gliwice, Poland). High-purity deionized water (resistivity of 18.2 MWcm), obtained using an Ultrapure Millipore Direct-Q-R 3UV (Millipore, Bedford, MA, USA), was used throughout the analysis. Professional polypropylene recipients certified for trace elements analysis (DigiTubes and DigiFilters), which were applied for the digestion, filtration and digest storage, were purchased from SCP SCIENCE, Canada.

### 2.2. Materials

#### 2.2.1. Dietary Supplements

In total, 9 dietary supplements containing various forms of Se were used in the study. Samples of dietary supplements were selected to represent the most popular chemical forms of the tested elements, on the basis of popularity in the largest chain pharmacies in Poland. The inclusion criteria were as follows: dietary supplements—a “DS” category of products, wide availability on the pharmaceutical market, products within the expiry date. The following exclusion criteria were adopted: difficult accessibility during the research, over-the-counter (OTC) category of products. Because the purpose of the research was not to assess the quality of individual products and their producers, trade names of individual supplements were not provided, but were only marked with numbers. The exact characteristics of the supplements used in the study are presented in Table 1.

For each product, three subsamples were taken from three different series. All determinations were made in triplicates. The study was conducted in 2021–2022. In total, the bioavailability of Se was evaluated in 30 different experimental models, considering the number of dietary supplements and the diet types used in the study. For each experimental model, three separate samples were evaluated, so the bioavailability of Se was determined in 90 analytical samples.

#### 2.2.2. Reconstructed Diet Duplicates

Diet is a special way of human nutrition based on the principles of dietetics and proper nutrition, and it is characterized by a fixed composition of consumed food in terms of quality, quantity and variety. The established daily food ration is characterized in terms of the type and quantity of individual food products and thus is characterized by a specific nutritional value, i.e., the content of energy and individual nutrients. The diet provides the nutrients necessary for the proper functioning of the body. However, a complicated matrix of the food taken during meal consumption influences the absorption of particular nutrients, in a positive or negative way [47]. Therefore, in order to evaluate the bioavailability of Se in the presence of various types of diets, three different types of diets—the most frequently observed in human nutrition—were developed based on the professional dietary scientific literature and the experience of a professional dietician (KI) [47,48,49], reconstructed and used during scientific experiments in the present study. Three types of human diets were developed and reconstructed—standard, basic and high-residue diets. The nutritional value of each diet was calculated using Dieta 6.0 Software (National Food and Nutrition Institute, Warsaw, Poland). The exact composition of diets and their nutritional value (including analytically determined content of Se) are presented in the Supplementary Material (Tables S1 and S2). Briefly, the content of proteins was 116.8, 153.9 and 143.5 g for basic, standard and high-residue diets, respectively. The high-residue diet was characterized by the highest carbohydrate content, 416 g, while the basic and standard diets contained 338 and 272.2 g, respectively. Fat content was 109.2, 129 and 117.1 g for basic, standard and high-residue diets, respectively.

All products used to prepare reconstructed DFRs came from the retail market of the Lublin region. Due to the fact that it is an agricultural region, over 90% of the most important products (cereal products, milk and milk products, meat and meat products, eggs, vegetables and fruit) were of local origin. The dishes were prepared and cooked in the laboratory in a manner similar to local culinary practices, according to the guidelines described in the available literature and using a procedure that was previously checked and validated [5,6,50,51]. In accordance with the international WHO/GEMS/Food recommendations, the food was prepared using drinking water from the city where the food products were purchased, in this case, Lublin. Salt and spices were added in the lab as in common cooking practices. Appropriate stainless steel equipment was used for food preparation, and aluminum, ceramic or enamel containers and utensils were avoided. The reconstructed diets were stored in plastic containers. The daily food rations included portions of main meals identical to those consumed by the individual, as well as other foods and beverages consumed daily (including tea and coffee). Inedible parts (bones and fish bones) were removed. Equipment used for the preparation and homogenization of composite samples was thoroughly cleaned before each preparation (e.g., cleaning with lab-grade detergent, thorough rinsing with hot tap water, rinsing or soaking in acid solution, thorough rinsing with deionized water) to avoid the risk of cross-contamination. The combined diets were weighed and homogenized in a homogenizer (Zelmer, Poland) with titanium blades and stored at −20 °C prior to analysis.

### 2.3. Two-Phase Enzymatic Model of In Vitro Digestion

In the present work, one of the first etching models developed by Miller et al. [52], with subsequent modifications, was used. It is a two-phase scheme of gastro-small intestine digestion with the use of appropriate digestive enzymes, while maintaining the appropriate temperature and pH of the system in which the enzymes can be active. The basic method was improved, especially using cellulose dialysis tubes, which better simulate natural conditions in the digestive tract [16].

Prior to the start of the first stage of digestion, 25 g of the diet homogenate was weighed and filled with deionized water to 50 g. In the model in which dietary supplements were added, the situation was comparable. Twenty-five grams of the diet homogenate was weighed, a portion of the dietary supplement equal to one serving was added and the whole system was filled with deionized water to attain a mass of 50 g.

Before starting the gastric digestion simulation, the entire system was acidified to pH = 2 using a 2 mol/L HCl solution. After the desired pH was reached, 2 mL of 10% pepsin in 0.1 mol/L HCl was added to each of the samples. The samples were tightly closed and placed for 2 h in a thermostatic water bath with a shaker (Vibra, AJL electronic, Poland) at 37 °C. Upon completion of this step, the pH of the sample solutions was changed from 2 to 6.5 using a 6% NaHCO_3_ solution. After the appropriate pH value (6.5) was obtained in each system, 5 mL of 0.4% pancreatin in 0.1 mol/dm^3^ NaHCO_3_ was added to each sample. From this moment, the second stage of digestion, namely the intestinal digestion, began. The samples adjusted to the desired pH (6.5) and treated with pancreatin were quantitatively transferred to cellulose dialysis tubes, which had previously been soaked for 12 h in 0.1 mol/mL HCl, and then rinsed several times with deionized water. The dialysis tubes were sealed and placed in a polypropylene container with 500 mL of deionized water and were then shaken for 2 h in a thermostatic water bath with a shaker at a temperature of 37 °C. After intestinal digestion, two fractions were obtained and subjected to further analysis: the dialysate, i.e., the solution surrounding the cellulose dialysis tube, and the residue in the cellulose dialysis tube. Simultaneously, under the same conditions, an analysis of the control samples was performed.

**Figure 1:**
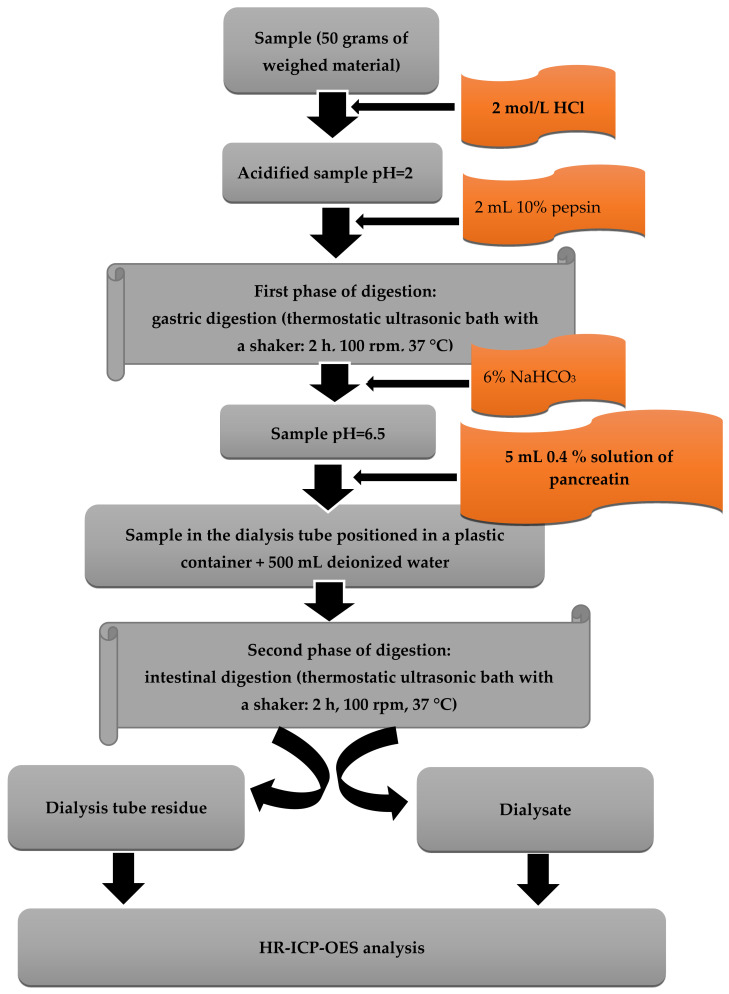
Schematic illustration of the digestion procedure.

A schematic illustration of the digestion protocol is presented in Figure 1.

### 2.4. Analytical Determination of Se

The obtained samples were digested using a wet digestion process with DigiPREP MS (SCP Science, Canada) equipment with a condensate recirculation system. The dialysate solutions (5 mL) were digested using 1 mL of 65% HNO_3_ for 120 min at the temperature of 120 °C, whereas 3 mL of 65% HNO_3_ was added to the cellulose dialysis tube residue (1 g) and left for 24 h. Then, 1 mL of 30% H_2_O_2_ was added, and the whole process was performed for 120 min at the temperature of 120 °C. After the digest solutions reached room temperature, they were filtered using a Rocker 300 vacuum pump (RockerScientific, New Taipei City, Taiwan) and DigiFILTER filters and then filled with deionized water to a final volume of 10 mL. In the obtained solutions, Se was determined using the ICP-OES method with a PlasmaQuant 9000 Elite, a high-resolution inductively coupled plasma optical emission spectrometer (Analityk Jena, Jena, Germany). The determinations were performed in triplicates. Instrumental settings are presented in Table S3 in the Supplementary Material. Appropriate dilutions of PlasmaCAL (SCP SCIENCE, Canada) Se standard solutions (1000 µg/mL) were used for the calibration of the equipment.

The applied methodology had already been validated and checked for its accuracy and precision in the determination of trace elements, including Se [6,53]. However, due to the use of a different method of mineralization, it was checked once again in terms of its suitability for the determination of selected elements in the studied samples. Therefore, a mixture of flour and milk powder (7:3 *w*/*w*) was fortified with known concentrations of various elements, including Se. The analysis of both the samples and reference material was performed simultaneously under the same conditions. Six replicates were performed during reference material determinations, and the obtained results are shown in Table 2.

### 2.5. Calculation of the Obtained Data

One of the aims of the research work was to assess the relative bioavailability of the studied elements from the diet homogenate with the addition of a dietary supplement in the in vitro dialysis model of digestion. The relative bioavailability of the studied elements varied depending on the diet used, the chemical form present in the preparation and the pharmaceutical form in which the dietary supplements used in the studies were present.

Se bioavailability ratios, expressed as percentages, were calculated using the following equation:

$$B\%=\left(\frac{D+Dr}{T+D}\right)\cdot 100\%$$

where B%—the percentage of the bioavailability (relative bioavailability) of Se, D—the amount of the element (mg) in the dialysate, Dr—the amount of the element (mg) corresponding to the equilibrium of concentrations on both sides of the cellulose membrane present inside the dialysis tube, T—the amount of the element (mg) present in the digest of the dialysis tube residue. Dr was calculated using the following equation:

$$Dr=\frac{\left(Cd-Ck\right)\cdot Vt\cdot R}{1000}$$

where Cd—the concentration of Se in the dialysate solution (μg/mL), Ck—the concentration of Se in the control sample (μg/mL), Vt—the volume of the dialysis tube (mL), R—the dilution factor.

**Figure 2:**
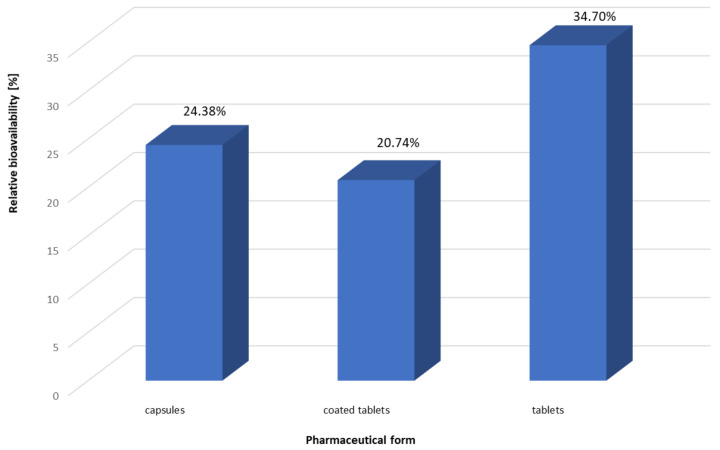
Bioavailability of Se considering the pharmaceutical form of the dietary supplements.

In general, obtained results of Se bioavailability were measured and developed in the following ways:

- Bioavailability of Se in three diets—baseline;
- Bioavailability of Se from nine dietary supplements under the influence of three diets (Table 3);
- Influence of chemical forms of supplements under the influence of three diets on the bioavailability of Se (Table 4);
- Influence of pharmaceutical forms of supplements (capsules, coated tablets, tablets) on the bioavailability of Se under the influence of three diets (Figure 2).

### 2.6. Statistical Analysis

All data obtained during the research were statistically processed using the computer program Statistica v. 13.0 (StatSoft, Kraków, Poland). MS Excel 2010 was used to collect data and support statistical analyses (Microsoft). In order to present the obtained results on a quantitative scale, descriptive statistical methods were used, i.e., arithmetic mean (x, M), median (Me), standard deviation (SD), quartile range (IQR), minimum (Min), maximum (Max), and F and H test statistic values for the ANOVA test. The Shapiro–Wilk test was used to evaluate the compliance of the distribution of the examined variables with the normal distribution. When the normal distribution of variables was demonstrated, parametric tests were used; in the absence of normal distribution, non-parametric tests were used.

The analysis of variance, one-way ANOVA with Tukey’s post hoc test, was used to assess statistically significant differences between many groups in the case of analyzing data on the relative bioavailability of elements from dietary supplements and medicinal products. One-way ANOVA with Tukey’s post hoc test and Kruskal–Wallis rank with Dunn’s post hoc test were used to assess differences between multiple groups when analyzing the impact of chemical forms of elements contained in dietary supplements and medicinal products, as well as forms of the preparations used, on the value of relative bioavailability. A value of 0.05 was assumed (α = 0.05) as the critical significance level (α) for all tests. Based on the obtained results, the following levels were set: *p* < 0.05—statistical significance; *p* < 0.01—strong statistical significance; *p* < 0.001—very strong statistical significance.

**Table 3:** Bioavailability of Se from dietary supplements under the influence of various types of diets.

Dietary Supplement No.	Chemical Form	Diet	M	Me	Min	Max	IQR	SD	One-Way ANOVA		Tukey’s Post Hoc Test Results		
									F	*p*	Group 1	Group 2	*p*
Without (%)	---	Standard	49.12	49.95	43.82	51.86	4.13	2.85	6.32	<0.001	Standard	Basic	>0.05
		Basic	47.62	47.29	45.73	51.58	1.30	1.70			Standard	High-residue	<0.01
		High-residue	44.81	44.13	40.53	49.61	5.16	3.07			Basic	High-residue	>0.05
1	sodium selenite	Standard	25.83	25.25	24.33	27.78	2.06	1.22	1.17	>0.05	Standard	Basic	>0.05
		Basic	25.45	27.09	21.18	29.72	4.62	3.16			Standard	High-residue	>0.05
		High-residue	24.45	24.69	23.78	25.12	0.81	0.51			Basic	High-residue	>0.05
2	sodium selenite	Standard	20.03	19.96	19.05	21.03	1.58	0.79	1.47	>0.05	Standard	Basic	>0.05
		Basic	19.31	19.33	18.29	20.32	1.05	0.69			Standard	High-residue	>0.05
		High-residue	19.68	19.93	17.97	21.40	1.36	1.15			Basic	High-residue	>0.05
3	sodium selenite	Standard	19.13	18.84	16.09	22.34	2.12	2.17	50.60	<0.001	Standard	Basic	<0.001
		Basic	22.59	22.33	20.80	24.38	1.75	1.14			Standard	High-residue	<0.001
		High-residue	15.82	15.83	15.36	16.28	0.47	0.34			Basic	High-residue	<0.001
4	sodium selenite	Standard	20.21	20.15	16.06	24.27	1.01	2.14	59.26	<0.001	Standard	Basic	<0.001
		Basic	27.80	27.94	26.73	28.56	1.04	0.64			Standard	High-residue	<0.05
		High-residue	22.05	22.39	19.69	24.42	0.97	1.47			Basic	High-residue	<0.001
5	selenized yeast SelenoPrecise	Standard	26.12	26.21	24.51	27.74	1.72	1.17	19.09	<0.001	Standard	Basic	<0.001
		Basic	29.80	29.82	28.69	30.88	1.36	0.77			Standard	High-residue	>0.05
		High-residue	25.81	25.99	22.57	29.05	3.07	2.24			Basic	High-residue	<0.001
6	selenized yeast	Standard	23.24	24.05	18.62	27.87	4.62	3.20	114.34	<0.001	Standard	Basic	<0.001
		Basic	36.33	36.01	35.69	37.55	0.77	0.63			Standard	High-residue	>0.05
		High-residue	24.50	24.56	22.58	26.42	1.56	1.29			Basic	High-residue	<0.001
7	sodium selenite	Standard	24.19	24.21	21.97	26.41	2.34	1.64	161.64	<0.001	Standard	Basic	<0.001
		Basic	30.36	29.96	29.50	31.32	1.32	0.77			Standard	High-residue	<0.001
		High-residue	21.50	21.39	20.93	22.08	0.56	0.40			Basic	High-residue	<0.001
8	selenized yeast	Standard	26.11	26.31	20.50	30.18	4.25	3.02	10.99	<0.001	Standard	Basic	<0.01
		Basic	22.44	22.37	21.59	23.29	1.20	0.65			Standard	High-residue	<0.001
		High-residue	22.00	21.88	19.77	24.22	3.38	1.73			Basic	High-residue	>0.05
9	sodium selenate	Standard	46.18	46.14	44.58	48.07	1.02	1.11	1035.02	<0.001	Standard	Basic	<0.001
		Basic	66.10	66.09	64.69	67.42	0.88	0.93			Standard	High-residue	<0.001
		High-residue	62.28	62.19	61.05	63.79	0.76	0.90			Basic	High-residue	<0.001

F—test statistic value for the ANOVA test; *p*—statistical significance.

**Table 4:** Bioavailability of Se considering chemical form under the influence of various types of diets.

Chemical Form	Diet	M	Me	Min	Max	IQR	SD	One-Way ANOVA		Tukey’s Post Hoc Test Results		
								F	*p*	Group 1	Group 2	*p*
Sodium selenite (%)	Standard	21.88	21.03	16.06	27.78	4.87	3.11	19.21	<0.001	Standard	Basic	<0.001
	Basic	25.10	26.73	18.29	31.32	6.84	4.20			Standard	High-residue	>0.05
	High-residue	20.70	21.32	15.36	25.12	3.81	3.03			Basic	High-residue	<0.001
Sodium selenate (%)	Standard	46.18	46.14	44.58	48.07	1.02	1.11	1035.02	<0.001	Standard	Basic	<0.001
	Basic	66.10	66.09	64.69	67.42	0.88	0.93			Standard	High-residue	<0.001
	High-residue	62.28	62.19	61.05	63.79	0.76	0.90			Basic	High-residue	<0.001
Selenized yeast (%)	Standard	24.67	25.11	18.62	30.18	6.24	3.36	8.35	<0.001	Standard	Basic	<0.05
	Basic	29.38	29.49	21.59	37.55	13.64	7.17			Standard	High-residue	>0.05
	High-residue	23.25	23.72	19.77	26.42	2.68	1.96			Basic	High-residue	<0.001
Selenized yeast SelenoPrecise (%)	Standard	26.12	26.21	24.51	27.74	1.72	1.17	19.09	<0.001	Standard	Basic	<0.001
	Basic	29.80	29.82	28.69	30.88	1.36	0.77			Standard	High-residue	>0.05
	High-residue	25.81	25.99	22.57	29.05	3.07	2.24			Basic	High-residue	<0.001

F—test statistic value for the ANOVA test; *p*—statistical significance.

## 3. Results

### 3.1. Influence of the Diet on the Bioavailability of Se from Dietary Supplements

Table 3 presents the results of the bioavailability of Se from dietary supplements under the influence of various types of diets (standard, basic and high-residue) used in the study. The relative bioavailability of Se from various types of diets (without addition of dietary supplements) was within the range of 44.81–49.12%. The highest relative bioavailability of Se was found in the standard diet (49.12%), and the lowest was found in the high-residue diet (44.81%); these differences were statistically significant (*p* < 0.01). There were no statistically significant differences in the relative bioavailability of Se between the standard and basic diets.

Focusing on particular dietary supplements, the relative bioavailability of Se within the groups varied. In the case of supplement No. 1, no statistically significant differences in the bioavailability of Se between the studied types of diets were found. The results obtained for supplement No. 2 also showed no statistically significant differences in the relative bioavailability of Se between various types of diets. In the case of product Nos. 3 and 4, the results for each of the diets showed statistically significant differences in the relative bioavailability of Se. In general, all these four dietary supplements contained Se in the form of sodium selenite; all were vitamin–mineral products with a complicated composition, and the determined bioavailability of Se from these products varied in the range of 15.82–27.80%, depending on the products and types of the diets used in particular models. In the studies using supplement No. 7, which also contained sodium selenite, statistically significant differences in the relative bioavailability of Se were observed. The mean results were 21.50–30.36%. In detail, a statistically significantly lower relative bioavailability of Se from the standard diet compared to the basic diet (*p* < 0.001) was revealed. In the case of the standard diet and the basic diet, a statistically significantly higher relative bioavailability of Se was demonstrated compared to a high-residue diet (*p* < 0.001) in the experiments using this product.

### 3.2. Influence of the Diet and Chemical Form on the Bioavailability of Se

Se in the dietary supplements used during the experiments was present in four chemical forms: sodium selenite, sodium selenate, selenized yeast and registered compound selenized yeast SelenoPrecise. Each of these chemical forms was analyzed considering its impact on the relative bioavailability of Se in the presence of each diet used in the study. The obtained results were presented in Table 4.

The majority of dietary supplements available on the market contain Se in the form of sodium selenite, which contains Se in the fourth oxidation state. The average results of the relative bioavailability of Se in that chemical form were within the range of 20.70–25.10%, depending on the type of diet used in the experimental model. A statistically significantly higher relative bioavailability of Se from the basic diet was demonstrated compared to both the standard diet (*p* < 0.001) and the high-residue diet (*p* < 0.001). On the other hand, there were no statistically significant differences in the relative bioavailability between the standard and high-residue diets for Se in the form of sodium selenite.

For sodium selenate (VI), the average results were 46.18–66.10%. A statistically significantly lower relative bioavailability of Se from the standard diet was shown compared to both the basic diet (*p* < 0.001) and the high-residue diet (*p* < 0.001). Moreover, significantly higher results were obtained for the basic diet compared to the high-residue diet (*p* < 0.001).

In the case of Se-enriched yeast, the average results of the relative bioavailability of this element were 23.25–29.38%. The studies performed revealed a statistically significantly higher relative bioavailability from the basic diet compared to both the standard diet (*p* < 0.05) and the high-residue diet (*p* < 0.001). There were no statistically significant differences in the relative bioavailability of Se in that form between the standard and high-residue diets.

For Se-enriched yeast in the form of the registered SelenoPrecise formula, the average relative bioavailability results for this element were 25.81–29.80%. For this chemical formula, a statistically significantly higher relative bioavailability from the basic diet, compared to both the standard diet (*p* < 0.001) and the high-residue diet (*p* < 0.001), was shown. There were no statistically significant differences between the standard and high-residue diets.

To present a more general look at the issue of the influence of the chemical form on the bioavailability of Se, all obtained data were averaged without a breakdown by type of diet. These data are presented in Table 5.

Sodium selenate was characterized by the highest bioavailability, which on average reached 58.19%. In contrast, the average bioavailability of sodium selenite, the form of Se most commonly used in the dietary supplements, was only 22.56% and was the lowest of all chemical forms of this element evaluated in the present study.

A statistically significantly lower relative bioavailability of Se was revealed for sodium selenite compared to experiments using the chemical form of Se-enriched yeast SelenoPrecise (*p* < 0.001), Se-enriched yeast (*p* < 0.05) or sodium selenate (*p* < 0.001) and compared to experiments without a dietary supplement (*p* < 0.001). Moreover, the results for SelenoPrecise were also statistically significantly lower compared to experiments using the chemical form of sodium selenate (*p* < 0.001) and compared to experiments without a dietary supplement (*p* < 0.01), where the natural bioavailability of Se from the diet was evaluated. It should be noted that a statistically significantly lower relative bioavailability of Se was also demonstrated in experiments using the chemical form of Se-enriched yeast compared to experiments using the chemical form of sodium selenate (*p* < 0.001) and compared to the bioavailability of Se from the diet (without addition of dietary supplement) (*p* < 0.001). There were no statistically significant differences between the remaining chemical forms of Se, and the average results of the relative bioavailability of Se from these systems oscillated within the range of 22.56–27.24%. In general, results of the relative bioavailability of Se in the form of sodium selenate were by far much higher in comparison to other chemical forms of this element. It should be emphasized that the results of the present research revealed a very high bioavailability of Se from a natural diet, which was lower only than the results for sodium selenate. However, these differences were statistically insignificant.

### 3.3. Influence of the Pharmaceutical Form on the Bioavailability of Se

An additional aim of the conducted research was to assess the bioavailability of Se considering the pharmaceutical form of the dietary supplements used in the study. The obtained results are presented in Figure 2.

In the current study, the dietary supplements used in the experiments were in three pharmaceutical forms: capsules, coated tablets and tablets. The obtained results of the relative bioavailability were evaluated considering the type of pharmaceutical form of particular dietary supplements that were used in the models in which they were mixed with an appropriate type of diet. It was revealed that the highest value of this parameter (average value for all diets used in the study) was obtained in the case of tablets (34.70%), and the lowest was obtained in the models with the use of dietary supplements in the form of coated tablets (20.74%). The bioavailability of Se from tablets was statistically significantly higher in comparison to capsules and coated tablets (*p* < 0.001). Moreover, it was revealed that a statistically significantly higher relative bioavailability of Se in the presence of a diet was observed in experiments using capsules, compared to coated tablets (*p* < 0.001). No statistically significant differences were observed between the capsules and the coated tablets.

## 4. Discussion

For many people and societies, Se requirements cannot be met from natural sources, and additional supplementation is needed. In such a situation, the use of dietary supplements is one possible solution for providing this element [54]. Niedzielski et al. [54] conducted research on the bioavailability of Se from commercially available dietary supplements on the Polish market, but the current study is the first in which the bioavailability of Se was evaluated in the presence of a complex food matrix, which more reliably reflects the conditions of taking dietary supplements in reality. Additionally, a comparison between various pharmaceutical forms was performed, which was also not previously performed for the dietary supplements containing Se. In the present study, various dietary supplements were evaluated in terms of the bioavailability of their Se content under the influence of various types of diets. In the studies on the influence of chemical forms of Se on its bioavailability, both organic and inorganic forms were compared. It has been shown that organic forms of Se, with Se-enriched yeast as the carrier, are better absorbed than inorganic forms [22]. In the present study, however, the highest relative bioavailability was revealed for sodium selenate (VI). A similar trend was demonstrated in the studies conducted by Zagrodzki et al. [44], who also obtained the highest relative bioavailability value for the chemical form of selenate (VI). Although the routes of Se absorption have not yet been fully described and characterized [39], it seems that Se in the form of selenate or selenite is very well absorbed, but less retained in the body compared to its organic forms, such as selenomethionine or selenocysteine [55]. Thus, the effective absorption value may be even higher for inorganic forms, but a true retention in, and a final release to, the body may be higher for organic forms. However, such an estimation can be performed only using in vivo models, as in vitro methods cannot estimate the amount of Se fraction that is actually retained in the body. According to Moreda-Piñeiro et al. [18,45] the bioavailability of Se in the form of selenomethionine (SeMet), which is the most common compound extracted from organic forms of selenium, was 55–80%, depending on the product and method used in the study. In the present research, a higher relative bioavailability of Se from organic forms compared to inorganic forms was not confirmed, and the obtained results for Se-enriched yeast were on average 25.77–27.24%, depending on the diet. The majority of studies evaluated the bioavailability of Se from single food products or dietary supplements without an additional food matrix. The bioavailability of Se is influenced by a complex foodstuff-matrix major composition, minor components and food processing. Results also depend on the method of evaluation and experimental conditions, which may have significantly influenced the final conclusions [45,56]. Moreover, the lower relative bioavailability of Se from Se-enriched yeast can be explained by the fact that the main source of Se in this case is the aforementioned selenomethionine, which is largely incorporated into the structure of proteins. Therefore, more complicated chemical combinations of Se may hinder its release, which in turn may affect the final values of the relative bioavailability of this element. Other compounds and chemical combinations of Se in yeast may also limit its relative bioavailability [44]. It should be noted, however, that the results obtained for inorganic forms were in agreement with scientific data suggesting that Se in the form of selenate (VI) is much better absorbed than in the form of selenite (IV) [45,56]. Importantly, despite showing higher values of relative bioavailability, Se in the form of selenate (VI) is not recommended due to its toxic properties [10], and this form is not so popular in the production of dietary supplements.

All the objectives of the current study were achieved. It was expected that the relative bioavailability of sodium selenate will be higher in comparison to sodium selenite, which was proved in the current study. However, it was also expected that the bioavailability of organic forms of Se will be higher in comparison to inorganic forms, which was not confirmed in the present study. The study revealed that the interactions between Se compounds and a complex matrix of the diet are very complicated. Previously, it was reported that dietary macro-components may significantly influence the bioavailability of Se. It was revealed that the bioavailability of Se is positively associated with the content of carbohydrates and fiber. In turn, protein content exhibits a negative correlation with the bioavailability of Se. So far, no correlation for dietary fat has been reported [32,46]. In the present study, it was observed that for the basic diet, in which the protein content was the lowest, the highest relative bioavailability of Se was determined. The same may be observed for the high-residue diet, in which the carbohydrate and fiber contents were the highest. For this type of diet, the obtained results were relatively high, considering particular dietary supplements as well as various chemical Se forms. In general, for the standard diet in which protein content was the highest and carbohydrate and fiber contents were the lowest, the lowest results of the bioavailability of Se were observed, which is in agreement with literature data, suggesting a similar influence of the macro-components on the bioavailability of Se. It was also noticed that the basic diet contained the lowest amount of fat, followed by the high-residue and standard diets, but more studies are needed to fully confirm that fat negatively influences the bioavailability of Se. In general, based on the results of the current study, it can be concluded that a balanced diet, containing products from all food groups, characterized by a moderate protein content and a high content of carbohydrates and fiber, could positively affect the bioavailability of Se from dietary supplements.

The study showed that the relative bioavailability of Se is affected by not only the chemical form or type of diet, but also the pharmaceutical form of dietary supplements. It is hard to find any literature data on the influence of the pharmaceutical form on the bioavailability of Se from dietary supplements; therefore, the obtained results preliminarily revealed that the highest bioavailability of Se was achieved for tablets, followed by capsules and coated tablets. Certainly, the obtained results depend to a large extent on the type and amount of additional substances used in the development of individual preparation formulations. More research is needed to fully confirm and unambiguously assess the effect of the pharmaceutical form on the bioavailability of Se.

It seems that the results obtained in this study may be of great practical importance in relation to the choice of dietary supplements by consumers, as well as for doctors in connection with the selection of the most appropriate products to supplement possible deficiencies of the element in the diet in the most effective way. In addition, the obtained data provide a practical hint regarding the influence of the type of diet on the bioavailability of Se, which may be helpful in the case of simultaneous use of supplements and food products.

The current study also exhibits some limitations. First, only nine dietary supplements were evaluated, and according to Zagrodzki et al. [44], in 2016 there were 614 dietary supplements containing Se on the Polish market, but only 296 products had any description of Se content. Moreover, the use of dietary supplements is very individual, as these products are used at different times of the day, with different meals and with different degrees of stomach filling. Therefore, in this study, typical diets used in nutrition were mapped to show the prevailing trends in how the composition of the food ration can affect the bioavailability of Se. Of note is the fact that the conclusions drawn from the performed studies were based on analytical determinations and thorough statistical analysis of the obtained data. Importantly, dietary supplements representing all chemical forms of Se used in the market and reconstructed food rations typical for the nutrition of healthy people were used in the study. It should also be remembered that the conducted analysis was based on the declaration of each producer regarding the chemical form of Se present in particular dietary supplements. Therefore, in the future, a speciation analysis may be needed to analytically check the actual chemical forms of Se in various products and to evaluate to what extent they are in agreement with producers’ declarations.

## 5. Conclusions

The performed studies revealed that the food matrix significantly influenced the bioavailability of Se from dietary supplements. It was revealed that sodium selenate was characterized by the highest value of this parameter, followed by organic forms and sodium selenite. The basic diet, characterized by moderate protein and high carbohydrate and fiber contents, positively influenced the bioavailability of Se. It was shown that the bioavailability of Se is also influenced by the pharmaceutical form of the product, and current determinations revealed that it was the highest for tablets, followed by capsules and coated tablets. To the best of the authors’ knowledge, this is the first study that characterizes, in an analytical way, complex interactions between the bioavailability of Se and dietary components, considering the chemical form of Se, the pharmaceutical form of the preparation and a thorough statistical analysis of the obtained data.

## Figures and Tables

**Table 1:** Detailed characteristics of dietary supplements used in the study.

	Se		
Product No.	Chemical Form	Supplement Type	Pharmaceutical Form
1	sodium selenite ^1^	vitamin–mineral	capsules
2	sodium selenite ^1^	vitamin–mineral	coated tablets
3	sodium selenite ^1^	vitamin–mineral	coated tablets
4	sodium selenite ^1^	vitamin–mineral	coated tablets
5	selenized yeast ^2^	multi-mineral	tablets
6	selenized yeast	single-mineral	tablets
7	sodium selenite ^1^	vitamin–mineral	tablets
8	selenized yeast	vitamin–mineral ^4^	capsules
9	sodium selenate ^3^	vitamin–mineral ^5^	tablets

^1^ Na_2_SeO_3_; ^2^ SelenoPrecise ^3^ Na_2_SeO_4_; ^4^ enriched with fish oil; ^5^ enriched with plant extracts.

**Table 2:** Selective parameters of the applied analytical determinations.

Parameter	Se (ICP-OES)
Reference value (mg/kg)	0.20
Determined value (mg/kg)	0.18
	0.18
	0.21
	0.19
	0.17
	0.16
**Average**	**0.18**
SD	0.017
RSD (%)	9.44
Recovery (%)	90.0
LOD (µg/kg)	2.20
LOQ (µg/kg)	7.50

SD—standard deviation; RSD—relative standard deviation; LOD—limit of detection; LOQ—limit of quantification.

**Table 5:** Average bioavailability of Se considering chemical form.

Chemical Form	M	Me	Min	Max	IQR	SD	Kruskal–Wallis ANOVA	
							H	*p*
Diet without supplement (%)	47.18	47.32	40.53	51.86	4.62	3.10	154.17	<0.001
Sodium selenite (%)	22.56	21.97	15.36	31.32	4.99	3.93		
Selenized yeast SelenoPrecise (%)	27.24	27.29	22.57	30.88	3.80	2.36		
Selenized yeast (%)	25.77	24.02	18.62	37.55	5.52	5.33		
sodium selenate (%)	58.19	62.19	44.58	67.42	19.25	8.85		

M—arithmetic mean; Me—median; Min—minimum; Max—maximum; IQR—quartile range; SD—standard deviation; H—test statistic value for the ANOVA test.

## Supplementary Materials

The following supporting information can be downloaded at: https://www.mdpi.com/article/10.3390/nu15061511/s1. Table S1: Composition of diets used in the study. Table S2: Selected nutritional parameters of diets used in the study. Table S3: Operating parameters in the ICP-OES method.
