# Supplementary material for: In Vitro Evaluation of Bioavailability of Se from Daily Food Rations and Dietary Supplements
Source: Nutrients. 2023 Mar 21;15(6):1511. doi: 10.3390/nu15061511 (PMC10058741; doi:10.3390/nu15061511)
Supplement: Supplementary file 1 [file nutrients-15-01511-s001.zip › nutrients-2290694-supplementary.pdf]

# SUPPLEMENTARY FILE

**Table S1.** Composition of diets used in the study.

| Type of the meal | Food product/meal | Amount (g/mL) |
|---|---|---|
| **Basic diet** | | |
| Breakfast | cereal coffee with milk | 250 |
| | white bread | 80 |
| | cottage cheese | 110 |
| | Onion | 10 |
| | cucumber | 30 |
| | butter | 10 g |
| Second breakfast | mixed bread (wheat-rye flour) | 60 |
| | chicken pate | 130 |
| | apple | 150 |
| | black tea infusion | 250 |
| Lunch | vegetable soup (including carrots, celery, parsley, cauliflower) | 400 (vegetables – 100 g) |
| | poultry chop | 150 |
| | potatoes | 300 |

| | | |
|---|---|---|
| | red cabbage salad | 150 |
| | grated strawberry compote | 250 (strawberries -30 g) |
| Dinner | meatballs in sauce | 120 |
| | pasta | 120 |
| | salad (carrot, apple, mayonnaise) | 100 |
| | yeast cake with crumble | 50 |
| | black tea infusion with milk | 250 |
| **Standard diet** | | |
| Breakfast | ham sausages | 150 |
| | mixed bread (wheat-rye flour) | 90 |
| | cocoa with milk | 250 |
| | mustard | 20 |
| Second breakfast | gouda cheese | 60 |
| | crispbread | 30 |
| | coffee infusion with milk | 150 |
| Lunch | tomato soup with pasta | 400 |
| | Potatoes | 300 |
| | grilled cod | 200 |

| | | |
|---|---|---|
| | salad with sauerkraut | 150 |
| | compote | 250 |
| | coffee infusion with milk | 150 |
| | milk chocolate with nuts | 30 |
| Dinner | mixed bread (wheat-rye flour) | 80 |
| | sausages | 110 |
| | pickled cucumber | 80 |
| | black tea infusion | 250 |
| **High-residue diet** | | |
| Breakfast | oatmeal in milk | oat flakes – 50 g<br>milk – 350 g |
| | wholemeal bread | 80 |
| | cottage cheese | 80 |
| | jam | 20 |
| Second breakfast | mixed bread (wheat-rye flour) | 80 |
| | rennet cheese | 40 |
| | ham | 440 |
| | tomatoes | 150 |

| | | |
|---|---|---|
| | banana | 100 |
| | black tea infusion | 250 |
| Lunch | beetroot soup | 400 (beetroot – 30 g; vegetables: carrots, parsley, celery – 25 g) |
| | pork chop | 150 |
| | potatoes | 300 |
| | boiled vegetables (carrots with peas 1+1 with breadcrumbs and butter) | 300 |
| | apple | 150 |
| | strawberry compote | 200 |
| Dinner | mixed bread (wheat-rye flour) | 80 |
| | chicken ham | 80 |
| | red pepper | 150 |
| | butter | 10 g |
| | coffee infusion with milk | 150 |

**Table S2.** Selected nutritional parameters of diets used in the study.

| Parameter | Diet | | |
|---|---|---|---|
| | **Basic** | **Standard** | **High-residue** |
| Proteins (g)<br>10-20% of E** | 116.8 (17.3% of E*) | 153.9 (22.2% of E) | 143.5 (18.5% of E) |
| Fats (g)<br>20-35% of E** | 109.2 (36.4% of E) | 129 (41.9% of E) | 117.1 (34% of E) |
| Carbohydrates (g)<br>45-65% of E** | 338 (46.3% of E) | 272.2 (35.8% of E) | 416 (47.5% of E) |
| Fiber (g) | 28.6 | 24.6 | 50.2 |
| Vitamin A (µg) | 2960 | 500 | 4600 |
| Vitamin C (µg) | 80.6 | 54.8 | 304.7 |
| Vitamin E (mg) | 17.2 | 15.9 | 24.7 |
| Calcium (mg) | 568.7 | 1203 | 1465 |
| Sodium (mg) | 2370 | 4865 | 3290 |
| Potassium (mg) | 4877 | 5615 | 6837 |
| Magnesium (mg) | 361 | 562.9 | 693 |
| Iron (mg) | 14.3 | 17.2 | 21.7 |
| Selenium (µg)<br>(determined value) | 41.5 | 45.7 | 42.3 |

| | | | |
|---|---|---|---|
| Energy (kcal) | 2699 | 2770 | 3099 |
| Total weight (g) | 2970 | 2750 | 3285 |

* Percentage of energy

** Recommended intake

**Table S3.** Operating parameters in the ICP-OES method.

| | |
|---|---|
| Analytical line, reading time | Se 196.028 nm, 6 s |
| Signal reading type | axial |
| Signal integration | 3 pix |
| Plasma generator power | 1300 W |
| Coolant gas flow rate | 14 $L\cdot min^{-1}$ |
| Auxiliary gas flow rate | 0.5 $L\cdot min^{-1}$ |
| Carrier gas flow rate | 0.6 $L\cdot min^{-1}$ |
| Sample flow rate | 1.0 $L\cdot min^{-1}$ |
